# Self-Calibrating TSEP for Junction Temperature and RUL Prediction in GaN HEMTs

**DOI:** 10.3390/nano15141102

**Published:** 2025-07-16

**Authors:** Yifan Cui, Yutian Gan, Kangyao Wen, Yang Jiang, Chunzhang Chen, Qing Wang, Hongyu Yu

**Affiliations:** 1 Engineering Research Center of Integrated Circuits for Next-Generation Communications, Ministry of Education, School of Microelectronics, Southern University of Science and Technology, Shenzhen 518055, China; 12131132@mail.sustech.edu.cn (Y.C.); 12333351@mail.sustech.edu.cn (Y.G.); 22112020122@m.fudan.edu.cn (K.W.); 11510044@mail.sustech.edu.cn (Y.J.); 2 Peng Cheng Laboratory, Shenzhen 518000, China; chenchzh@pcl.ac.cn; 3 School of Integrated Circuit, Shenzhen Polytechnic University, Shenzhen 518055, China

**Keywords:** GaN HEMTs, gate leakage current, R_DS_ON_, temperature-sensitive electrical parameter, remaining useful life

## Abstract

Gallium nitride high-electron-mobility transistors (GaN HEMTs) are critical for high-power applications like AI power supplies and robotics but face reliability challenges due to increased dynamic ON-resistance (R_DS_ON_) from electrical and thermomechanical stresses. This paper presents a novel self-calibrating temperature-sensitive electrical parameter (TSEP) model that uses gate leakage current (I_G_) to estimate junction temperature with high accuracy, uniquely addressing aging effects overlooked in prior studies. By integrating I_G_, aging-induced degradation, and failure-in-time (FIT) models, the approach achieves a junction temperature estimation error of less than 1%. Long-term hard-switching tests confirm its effectiveness, with calibrated R_DS_ON_ measurements enabling precise remaining useful life (RUL) predictions. This methodology significantly improves GaN HEMT reliability assessment, enhancing their performance in resilient power electronics systems.

## 1. Introduction

Enhancement-mode (E-mode) p-GaN gate GaN HEMTs have emerged in the consumer market and demonstrate great potential for AI power supplies and robotics [1,2,3,4,5,6]. However, they still face formidable reliability challenges, primarily due to the increase in R_DS_ON_ caused by current and voltage stresses in power circuits, which makes p-GaN HEMTs susceptible to electrical and thermomechanical failure. These failures account for 34% of all failures in GaN-based power systems [7,8,9].

R_DS_ON_ is a critical parameter for p-GaN HEMTs, as its increase often indicates exacerbated charge trapping and interface state alterations in the AlGaN/GaN channel, resulting in increased power consumption and reduced stability [10]. However, factors such as test time points, magnitude of V_BUS_, stress time, and device junction temperature all significantly impact R_DS_ON_ measurements. These influences can lead to inaccuracies in assessing the R_DS_ON_ increase due to device aging, impairing performance evaluation and remaining useful life (RUL) models [11,12,13].

Accurate monitoring of the junction temperature of GaN HEMT is critical for reliable R_DS_ON_ measurement and device reliability, as it is influenced by ambient temperature, duty cycle, and switching frequency [14,15]. There are four primary methods for measuring the junction temperature: optical, sensor-based physical contact, model-based thermal network, and temperature-sensitive electrical parameter (TSEP) methods [16]. The TSEP method is advantageous because of its ease of integration, fast response, and high accuracy, making it a superior choice compared with optical and physical contact methods, as well as model-based thermal network approaches [17,18]. For GaN HEMTs, gate leakage current (I_G_) is commonly used as a TSEP [19,20,21]. However, numerous studies indicate that I_G_ changes with HEMT aging [22,23,24], requiring calibration to maintain measurement accuracy, a gap not yet addressed in existing research.

To accurately measure the R_DS_ON_ of GaN HEMTs and utilize it for lifetime prediction, a self-calibrating TSEP approach is proposed. I_G_ is employed as a TSEP, precisely calibrated using physical models for the first time to ensure that the calibrated I_G_ can accurately estimate the operating junction temperature of the HEMTs, effectively mitigating the impact of gate aging on measurement results. This approach enables reliable R_DS_ON_ assessment and supports accurate RUL prediction for GaN HEMTs under diverse operating conditions.

## 2. Experiment Setup

A. Design of the Multiple-Pulse Tester board

**Figure 1:**
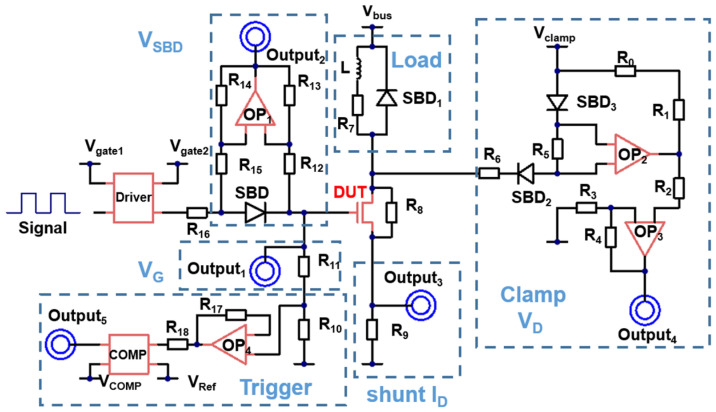
Schematic of the GaN HEMT evaluation board, illustrating the driving circuit, voltage-clamping circuit, and power supply/load circuit.

The unique high dV/dt (voltage slope) and dI/dt (current slope) events, along with the current oscillations induced by the load capacitance, present considerable challenges in the design of an efficient and dependable GaN HEMT test board. A conventional multiple-pulse tester generally comprises three primary components: a driving circuit, a voltage-clamping circuit, and a power supply/load circuit, as illustrated in the circuit schematic depicted in Figure 1 and Table 1.

The GaN HEMT test board incorporates several innovative designs to optimize the testing performance. An isolated gate driver IC (LMG1025-Q1) that effectively reduces gate interference and noise is utilized to ensure high accuracy. Instead of using resistors for gate-drive setting, a small signal Schottky diode (1N6263) is employed, which features a high breakdown voltage, a low turn-on voltage, and ultrafast switching. This diode, owing to its sensitivity to microampere-level currents, allows monitoring of I_G_ in the device by measuring the voltage across the diode. To determine the I_G_, the forward voltage drop (V_SBD_) across the Schottky barrier diode (SBD) connected to the DUT gate is measured using a differential amplifier. The relationship between V_SBD_ and I_G_ is precalibrated using an Agilent B1505A Power Device Analyzer (Agilent, Santa Clara, CA, USA). Due to the low operating current of the Schottky diode (1N6263), its aging effects are minimal, allowing a one-time calibration of the I_G_ before testing begins [25]. The power loop design incorporates a 1200 V SiC diode (SBD1) as a freewheeling diode.

For data acquisition and processing, the test board features a complete acquisition circuit including a V_G_ acquisition circuit (output1), a V_SBD_ acquisition circuit (output2), a V_DS_ON_ acquisition circuit (output3), and an I_DS_ON_ acquisition circuit (output4). The V_G_ acquisition circuit consists of a voltage follower and a comparator, with the comparator outputting the trigger signal (output5) for FPGA sampling. V_SBD_ acquisition is achieved through a differential amplification circuit that captures the voltage across the Schottky diode. The voltage acquisition clamp circuit has two SiC Schottky barrier diodes (SBDs). For current acquisition, a 0.1 Ω current shunt resistor (SSDN-414-10) is used. Throughout the acquisition circuit, high-speed operational amplifiers (LM6172IMX) minimize the sampling delay and noise, significantly enhancing the data accuracy and reliability. The sampling trigger signal is precisely synchronized with the rising edge of the gate signal to ensure accurate data collection.

In summary, these design choices collectively ensure repeatable measurements under harsh switching conditions, making the board suitable for accelerated aging tests.

B. Extraction of R_DS_ON_ and I_G_

In this study, a commercially available p-gate GaN HEMT (EPC2203, 80 V, 1.7 A rated Schottky-type p-gate GaN HEMT) was selected as the HEMT under test. The gate-source voltage (V_GS_) was set to 5 V in the on-state during the testing period.

**Figure 2:**
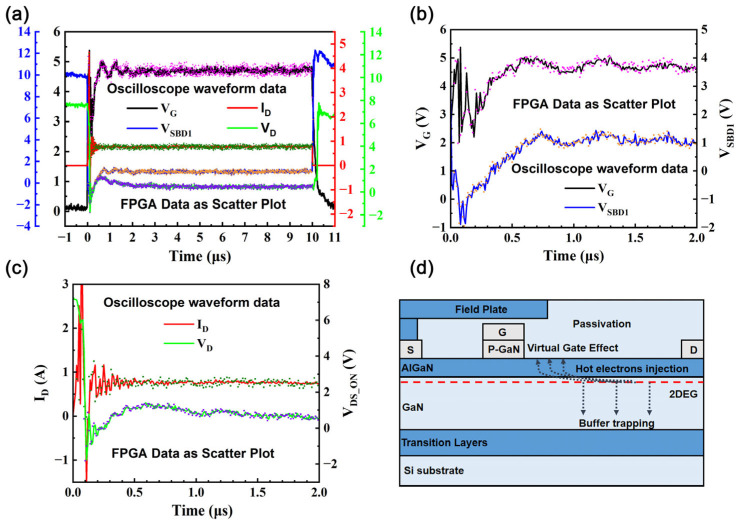
(**a**) Comparison of FPGA and oscilloscope measurements of hard switching test wave forms under V_bus_ = 80 V and T = 300 K. (**b**) V_G_ and V_SBD_ and (**c**) I_D_ and V_D_ wave forms during the first 2 µs after the device turns on. (**d**) Illustration of typical trapping locations that can lead to an increase in R_DS_ON_.

Figure 2 presents a comparative analysis of the hard switching test waveforms captured under 80V bus voltage (V_bus_) conditions using both an oscilloscope and the FPGA. The smooth transitions captured in the gate turn-on/off waveforms by both measurement methods provide compelling evidence of the optimized design of the gate drive circuit and power loop. Additionally, the system can derive I_G_ values from V_SBD_. There is high consistency between the FPGA measurement data and the waveforms recorded by the oscilloscope, with an error rate below 1%, as calculated from the data in Figure 2a.

R_DS_ON_ can be calculated based on the I_DS_ON_ and V_DS_ON_ values obtained during conduction. By adjusting the high-voltage power supply, R_DS_ON_ can be measured under various stress voltages. The R_DS_ON_ values were obtained under various bus voltages during the second turn-on pulse in the hard switching test. To ensure the accuracy of waveform analysis, the extraction time for R_DS_ON_ was set to the data points corresponding to 3 µs to 9 µs in the data packet. This window avoids transient effects and captures the steady R_DS_ON_.

**Figure 3:**
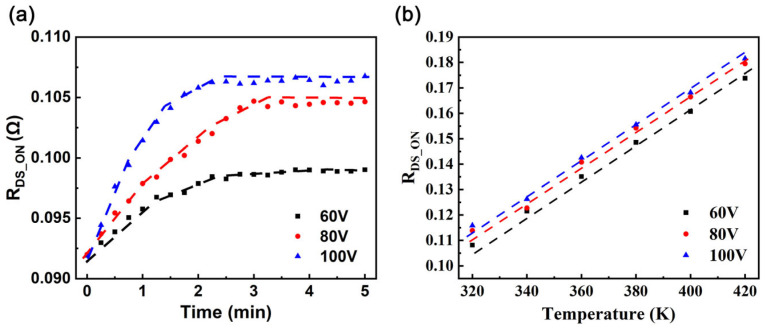
(**a**) R_DS_ON_ under continuous hard-switching stress at 300 K and a V_bus_ of 60 V, 80 V, and 100 V, with symbols representing experimental measurements and dashed lines indicating fitted data. (**b**) Junction temperature dependence of R_DS_ON_ at a V_bus_ of 60 V, 80 V, and 100 V, with symbols denoting measured values and dashed lines representing model fits.

To delve deeper into the specific impact of junction temperature on R_DS_ON_ and effectively eliminate its interference in subsequent measurements, a series of supplementary tests were conducted in this study. To minimize the interference caused by self-heating effects, two primary measures were adopted; first, the load resistance was increased from 100 ohms to 300 ohms, and second, the duty cycle of the HEMT was reduced to 10%. Shown as symbols for experimental measurements and dashed lines for model fits (Equation (1)) in Figure 3a, this presents the characteristics of the R_DS_ON_ variation during continuous hard switching under different V_bus_ values (60 V, 80 V, 100 V) at a junction temperature of 300 K. According to this chart, R_DS_ON_ significantly increases in the initial stage and subsequently stabilizes, reaching a saturated state. This saturated R_DS_ON_ is regarded as a reliable indicator of achievement of the steady-state R_DS_ON_ [23], which represents the dynamic equilibrium state reached by the capture and release processes within the HEMT. In the subsequent performance evaluations of the HEMT, all measured R_DS_ON_ values are treated as the saturated R_DS_ON_. To further analyze the impact of junction temperature, Figure 3b presents a comparison of the HEMT R_DS_ON_ values at different junction temperatures, while dashed lines show the model fits (Equation (1)), capturing the temperature-dependent behavior. The ASM-HEMT model accurately captures the temperature-dependent behavior of R_DS_ON_ by accounting for the mobility degradation of the 2DEG and the temperature sensitivity of access resistances, ensuring reliable simulations across a wide temperature range [26]. Considering that the conductivity of a two-dimensional electron gas decreases nearly linearly with increasing junction temperature and considering the lucky electron model [27,28], a simple empirical equation that relates R_DS_ON_ to the junction temperature and V_bus_ can be established, as shown in (1):

(1) $$R_{\mathrm{DS}\_\mathrm{ON}\;}=\;m(T-163)(1+n(V\;-80))R_{\mathrm{DS}\_\mathrm{ON}\_0}$$

where m and n are two parameters and R_DS_ON_0_ represent the R_DS_ON_ of the HEMT at 80 V and 350 K. Using (1), since R_DS_ON_ varies with operating conditions, each unique condition corresponds to a distinct RUL value. To enable consistent RUL prediction across diverse conditions, (1) normalizes all R_DS_ON_ measurements to the baseline condition (80 V, 350 K). By applying this calibration, the normalized R_DS_ON_ values isolate aging effects from operational variations, ensuring that RUL predictions are solely based on degradation trends rather than transient conditions.

## 3. Modeling and Calibration of the Temperature-Sensitive Parameter (I_G_) for HEMTs

In recent years, the I_G_ in normally on p-GaN HEMTs has become a subject of intensive research [9]. This parameter, which has been thoroughly characterized in previous publications, offers valuable insights into the electrical behavior of these HEMTs. One particularly fascinating aspect is the potential use of I_G_ as a TSEP.

**Figure 4:**
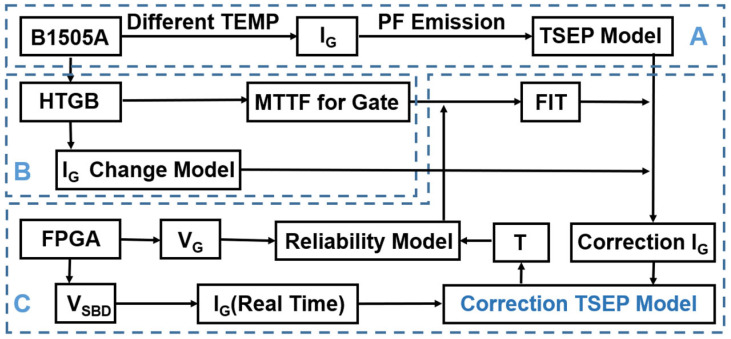
Block diagram of the modeling and calibrating temperature-sensitive parameters based on I_G_.

Figure 4 outlines a systematic methodology for modeling and calibrating TSEPs based on I_G_. The process begins with I_G_ testing conducted under various temperature conditions concurrently with a high-temperature gate bias (HTGB) test. Through I_G_ testing, the initial TSEPs are established. The HTGB test provides data on the expected mean time to failure (MTTF) of the HEMT gate under different conditions, as well as a model for the change in I_G_ with device aging. MTTF is the average time to failure derived from statistical analysis of multiple TTF (Time to Failure) values, where TTF represents the time until a single device fails. By subsequently integrating V_G_ data from relevant testing, junction temperature conditions, and the reliability model, based on the TTF derived from prior HTGB test results, we can estimate the failure in time (FIT) of the p-GaN gate. FIT is a statistical metric representing the expected number of failures per billion device-hours for a population. Finally, by correlating the p-GaN gate FIT with the I_G_ change model, we can calibrate I_G_ back to its initial state in the HEMTs. This calibration process enhances the long-term accuracy of I_G_ application in the TSEP model.

A. Operating Principle and Modeling of TSEPs for HEMTs

The core of the p-GaN HEMT gate structure lies in the Schottky-type configuration, which can be electrically represented by two back-to-back diodes. This complex structure leads to multiple mechanisms contributing to the gate leakage current, including thermionic field emission (TFE), Poole–Frenkel emission (PFE), trap-assisted tunneling, and Fowler–Nordheim tunneling [29,30,31,32,33,34]. The dominance of each mechanism depends on the specific bias and junction temperature conditions under which the HEMT operates. Under operating conditions with gate voltages above 2 V and junction temperatures exceeding 300 K, Poole–Frenkel emission (PFE) dominates the I_G_ behavior due to its strong temperature dependence and relevance to trap-assisted conduction in p-GaN HEMTs [31,34]. The PFE is a conduction mechanism where charge carriers are thermally excited from Coulombic traps within the material into the conduction band. The applied electric field lowers the effective potential barrier for this thermal emission by reducing the Coulombic energy barrier around the traps. Since this process relies on thermal energy to overcome the barrier, the carrier emission rate increases exponentially with junction temperature, following an Arrhenius-type dependence. Consequently, PFE becomes more prominent at higher temperatures, which explains its significance in modeling leakage current under elevated junction temperature conditions. Given the selected HEMT threshold voltage of approximately 1.7 V, a gate withstanding voltage of 5.5 V (as per the datasheet), and typical operating gate voltages ranging from 4 to 5 V, the PFE model was employed for fitting purposes. PFE can be described as follows [31,34]:

(2) $$I_{G}=\;AV_{G}\exp(\frac{-(B-\sqrt{CV_{G}})}{k_{B}T})\;$$

A, B, and C are constants, where A is associated with the trap density, B pertains to the trap level, and C relates to the dielectric constant. Additionally, k_B_ represents the Boltzmann constant, and T denotes the absolute junction temperature.

**Figure 5:**
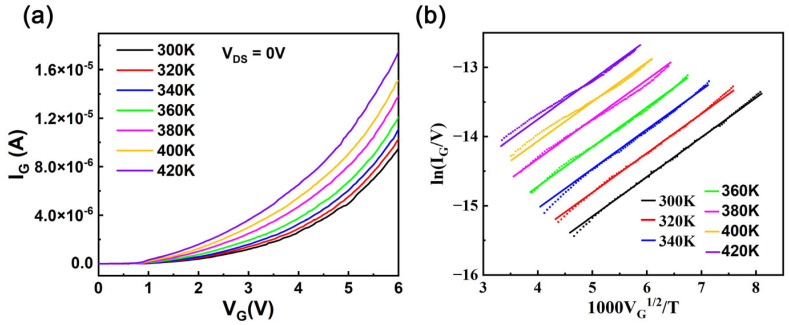
(**a**). I_G_−V_G_ characteristics of a fresh HEMT at junction temperatures ranging from 300 K to 420 K. (**b**). Plot of ln(I_G_/V) vs. 1000V_G_^1/2^/T under a V_G_ sweep from 2 V to 6 V, with solid lines representing measured data and dashed lines indicating fitted results from the PFE model.

The PFE characteristics are evident from the straight lines with similar slopes observed between 160 K and 440 K. In Figure 5b, solid lines represent experimental measurements of (ln(I_G_/V)) vs. (1000V_G_^1/2^/T), while dashed lines show fitted results from the PFE model. This comparison demonstrates that the gate leakage current is satisfactorily modeled by Equation (2).

**Figure 6:**
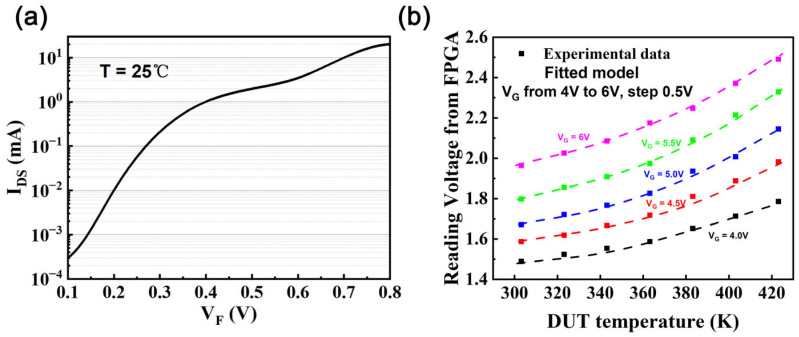
(**a**) Forward current of the gate SBD at T = 298K. (**b**) The SBD voltage drop at the gate as a function of the HEMT junction temperature at different values of the gate bias voltage, with solid lines representing measured data and dashed lines indicating fitted results from the Equation (3).

Test results for the gate SBD forward current, shown in Figure 6a, indicate that I_G_ ranges from 5 µA to 15 µA at V_G_ = 5 V for the GaN HEMT. The voltage drops across the gate SBD should be approximately 0.15 V to 0.25 V. The gain of the differential amplifier was set to 10. The final test results fall within the range of 1.5 V to 2.5 V. The junction temperature can be calculated using (3):

(3) $$T=\;\frac{-\sqrt{CV_{G}}}{k_{B}(\ln\left(\frac{I_{G}}{AV_{G}}\right)+B)}$$

The comparison between the fitted model and the actual data, as illustrated in Figure 6b, demonstrates good matching, indicating that under the specific test conditions (V_G_ = 5 V), the PFE model is an appropriate model for characterizing the gate leakage current in the p-GaN gate HEMT.

B. Modeling of I_G_ Variations and Lifetime Assessment of HEMT

High-Temperature Gate Bias (HTGB) tests are a critical method for evaluating the gate reliability and lifetime of High-Electron-Mobility Transistors (HEMTs). These tests apply a high static bias to the gate terminal to simulate aging effects and assess their impact on device performance, with a particular focus on gate leakage current (I_G_).

**Figure 7:**
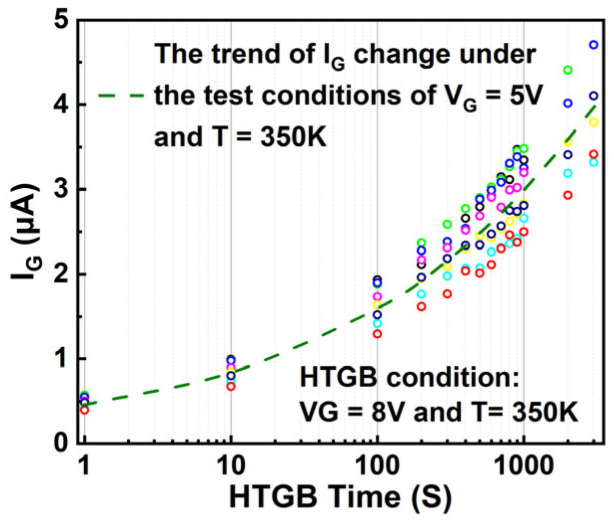
Time-dependent behavior of I_G_ at V_G_ = 5 V and T = 350 K during the HTGB (V_G_ = 8 V and T = 350 K) process, with points representing experimental measurements and dashed lines indicating fitted results from Equation (4). Each differently colored point set represents the aging-test results for an individual device.

The electrical measurements were performed using an Agilent B1505A Power Device Analyzer/Curve Tracer. HTGB tests were conducted at two junction temperatures, 350 K and 420 K, to study the effects of temperature on gate aging. Three gate voltages (V_G_) were applied—8.0 V, 8.5 V, and 9.0 V—to explore varying stress levels. Eight HEMTs were tested for each condition to ensure statistical reliability. During the tests, the drain and source electrodes were grounded to isolate the gate bias effects. Notably, I_G_ was measured periodically at V_G_ = 5 V and T = 350 K to consistently track its evolution under the HTGB stress conditions. Figure 7 presents the time-dependent behavior of I_G_, measured at V_G_ = 5 V and T = 350 K, during an HTGB test at V_G_ = 8 V and T = 350 K. The data points show experimental measurements of I_G_ taken at regular intervals, while the dashed lines represent the fitted results from Equation (4). This figure highlights a significant increase in I_G_—exceeding 1000%—as the gate ages, demonstrating that I_G_ is not a stable long-term temperature-sensitive electrical parameter (TSEP). This aging-induced variation in I_G_ has been overlooked in prior TSEP and condition monitoring studies, revealing a key gap that this research addresses.

The time-dependent breakdown (TDB) process escalates dielectric defects, and within the framework of the PFE mechanism, this higher defect count leads to a corresponding surge in I_G_ [35]. The generation of these defects exhibits power-law growth over time, t, and their formation is proportional not only to the energy of the injected carriers but also to the fluence [36]. As a result, Equation (4) accurately models the temporal variation of I_G_ under TDB conditions:

(4) $$I_{G}\;=\;\gamma {I_{G0}V}_{G}t^{\alpha \;}$$

where γ and α are treated as constants, and I_G0_ represents the initial gate leakage current of the HEMT under a specific V_G_. These constants, γ and α, were determined through curve fitting to experimental data on the time-dependent gate leakage current under stress conditions, ensuring the model accurately captures the observed behavior. The variable t denotes the duration of the applied stress. Importantly, temperature effects are not directly incorporated into this equation; instead, the subsequent reliability model (Equations (7) and (11)) converts the stress times at various junction temperatures into equivalent stress times at 350 K for consistency.

After the Aging-Dependent I_G_ change model, which captures the variation of I_G_ due to gate aging as described by Equation (4), is obtained, the I_G_ of the HEMT must be calibrated using its FIT rate to make I_G_ compatible with the TSEP model. To obtain the FIT, the initial step is to determine the MTTF of the HEMT under various gate operating conditions. This calculation uses the Weibull distribution, with the cumulative distribution function (CDF) from Equation (5) transformed to a log-log scale for analysis:

(5) log(−log(1−F(t)))=βlog(t)−βlog(η)

**Figure 8:**
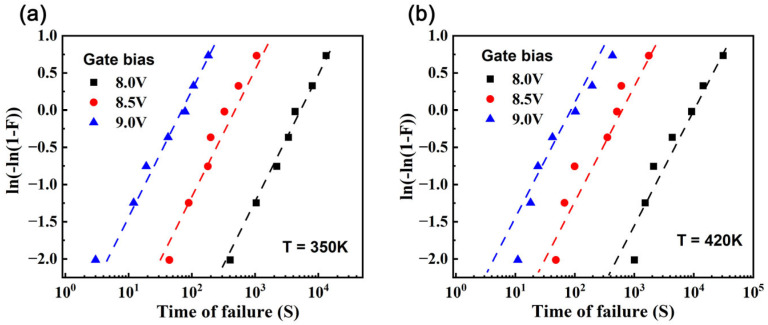
Weibull distribution chart for gate-source failure of GaN HEMTs at (**a**) T = 350 K and (**b**) T = 420 K, with points representing experimental failure data and dashed lines indicating fitted results from the Weibull distribution model (Equation (5)).

**Figure 9:**
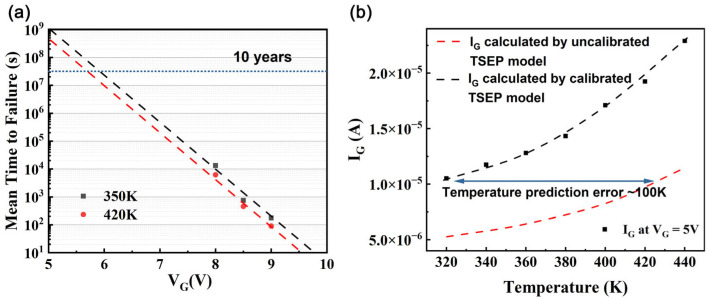
(**a**) MTTF for HEMTs versus VG at both 350 K and 420 K, with points representing experimental MTTF data and dashed lines indicating fitted results from Equation (6). (**b**) Comparison of the fitting results for the calibrated temperature-sensitive parameters and the uncalibrated parameters, with points representing experimental measurements and dashed lines showing fitted results from Equation (3) and calibrated TSEP model (Equation (12)).

From these transformed data, presented in Figure 8, the parameters β and η are extracted using linear regression. Specifically, β corresponds to the slope of the linear fit, and the intercept allows calculation of η through the relationship −βlog(η). These parameters are then substituted into the MTTF equation for the Weibull distribution:

(6) $$\mathrm{MTTF}=\eta \cdot \Gamma (1+\frac{1}{\beta })$$

where Γ represents the Gamma function. This approach enables precise MTTF calculation based on the Weibull distribution and parameters derived from Figure 9a. The estimated MTTF of the HEMTs at 350 K and 5 V is 1.3 × 10^9^ s.

Previous studies developed a physics-based lifetime model based on the impact ionization mechanism to explain the intrinsic wear-out process under gate bias, expressed as Equation (7) [36,37,38]:

(7) $$\mathrm{MTTF}=X/(1\;-\;C\Delta T)\;\exp[(Y/(1+V_{G}){)}^{1.9}]$$

where X and Y are constants, c = 6.5 × 10^−3^ K^−1^, and ΔT is the test junction temperature in units of Kelvin relative to 298 K. For the HEMT operating at V_G_ = 5 V in this work, the MTTF can be determined by substituting the extracted Weibull parameters into the MTTF formula.

C. TSEP Model Calibration Based on the Gate FIT and Aging-Dependent I_G_ Change Model

In the semiconductor industry, the FIT is a critical metric used to assess device reliability [38]. A FIT of one signifies one failure per 10^9^ device hours. Devices are subjected to various stress conditions, such as various operating durations and duty cycle variations, all of which impact their reliability. These conditions, which are influenced by factors such as voltage and junction temperature, result in distinct FIT values for each stress scenario. Directly summing FIT values from stress conditions like gate stress and channel stress (e.g., switching or reverse bias stress) can lead to inaccuracies due to interacting failure mechanisms. A more precise method calculates the total failure rate by weighting individual stress contributions, accounting for their duration and duty cycles, as detailed in the following equations. This can be mathematically expressed as [39]:

(8) $$\mathrm{FIT}={\mathrm{FIT}}_{1}+{\mathrm{FIT}}_{2}+\;\;...\;+{\mathrm{FIT}}_{i}$$

In this equation, the subscripts 1, 2 ... i are used to distinguish different stress conditions. The FIT is associated with a unique failure rate (FR_i_) under a specific stress condition that persists for a certain duration (t_i_). To calculate FITi, the following equation is employed:

(9) $${\mathrm{FIT}}_{i}={\mathrm{FR}}_{i}\;\times \;\;t_{i}$$

By substituting Equation (8) into Equation (9) and considering the duty cycle factor, the following formula for assessing the total failure rate under specific operating conditions and gate signals can be obtained:

(10) $$\mathrm{FR}=\frac{\;\mathrm{FR}1\;\times \;t_{1}}{t_{\mathrm{total}}}+\frac{\;\mathrm{FRi}\;\times \;t_{2}}{t_{\mathrm{total}}}+\ldots \;+\frac{\;\mathrm{FRi}\;\times \;t_{i}}{t_{\mathrm{total}}}$$

The FR can be replaced by 1/MTTF under the corresponding conditions. The working conditions of the device in our experiments are mostly close to V_G_ = 5 V and T = 350 K when the HEMT is turned on. Therefore, Equation (11) is generated:

(11) $$t_{\mathrm{total}}=(\frac{t_{1}}{{\mathrm{MTTF}}_{1}}+\frac{t_{2}}{{\mathrm{MTTF}}_{2}}+\ldots +\frac{t_{i}}{{\mathrm{MTTF}}_{i}}\;){\mathrm{MTTF}}_{{(V}_{G}=5V,T=350K)}$$

By integrating (3), (4), (7), and (11), a calibrated model for temperature-sensitive parameters is constructed. The detailed procedure is as follows:

1. Utilize Equation (7) to calculate the MTTF required in (11) for the specific operating conditions.
2. Utilize Equation (11) to calculate the equivalent stress time required in (4).
3. Utilize Equation (4) to calculate the origin I_G_.
4. Utilize Equation (3) to calculate the effect of junction temperature on I_G_.

Following these steps, the junction temperature of the device can be estimated through Equation (12):

(12) $$T=\frac{-\sqrt{CV_{G}}}{k_{B}(\ln\left(\frac{I_{G}}{A\gamma V_{G}^{2}e^{\alpha }((\frac{t_{1}}{{\mathrm{MTTF}}_{1}}+\frac{t_{2}}{{\mathrm{MTTF}}_{2}}+\ldots +\frac{t_{i}}{{\mathrm{MTTF}}_{i}}\;){\mathrm{MTTF}}_{{(V}_{G}=5V,T=350K)})}\right)+B)}$$

Figure 9b presents a comparison of the fitting results for the calibrated TSEPs and the uncalibrated TSEPs. After applying a stress of 8.5 V for 100 s, the error of the calibrated temperature-sensitive parameters remains within 1%, whereas the error of the uncalibrated model is approximately 30%, rendering it essentially ineffective. In practical work, each time the I_G_ and V_G_ are acquired via FPGA, the HEMT junction temperature is first calculated based on I_G_ and the existing TSEP model. The HEMT aging condition is subsequently further calibrated using Equation (7), followed by adjustment of the temperature-sensitive parameter model.

In summary, by incorporating the results of HTGB testing and I_G_ measurements at different V_G_ levels and junction temperatures, we developed a self-calibrating model. This model can be dynamically updated according to our test system, allowing precise calibration of the TSEP model.

## 4. Long-Term Test Results and RUL Prediction

Long-term reliability and stability of electronic components are paramount for their successful deployment across various applications. To further substantiate the efficacy of our TSEP model and evaluate the reliability of GaN HEMTs, we conducted extensive hard-switching tests. In these tests, the gate drivers operated at a fixed switching frequency of 50 kHz with a 50% duty cycle. This setup resulted in a current ranging from 0.6 A to 1 A flowing through an RL load with an impedance of 100 Ω. The gate voltage was varied between 0 V and 5 V.

**Figure 10:**
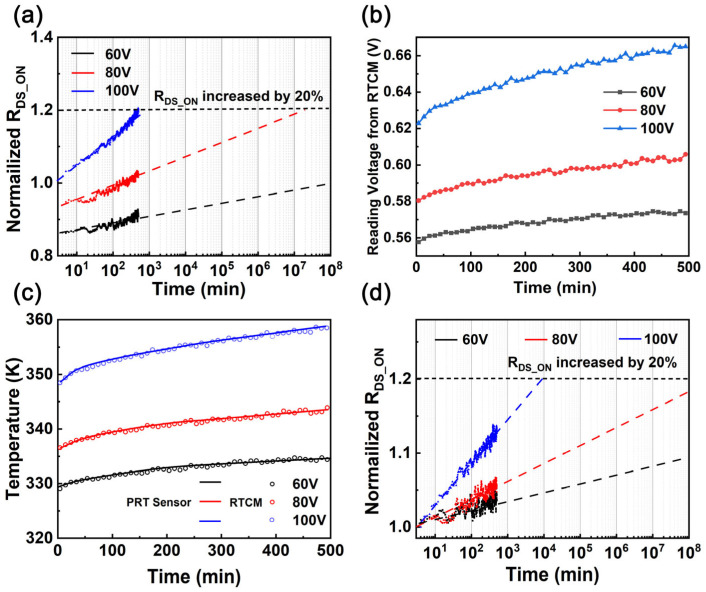
(**a**) R_DS_ON_ variation under three V_bus_ conditions of 60 V, 80 V, and 100 V over time. (**b**) V_SBD_ reading from the test system, with points representing experimental measurements and dashed lines indicating the R_DS_ON_ increase trend ignoring junction temperature changes. (**c**) Comparison of temperature measurements between the FPGA and PRT sensors. (**d**) Calibrated R_DS_ON_ variation under constant V_bus_ hard-switching conditions at equivalent settings of V_bus_ = 80 V and 350 K, with points representing experimental measurements and dashed lines showing fitted results from Equation (13).

Figure 10a visually presents the test results of R_DS_ON_ over time under three V_bus_ conditions: 60 V, 80 V, and 100 V. These results, obtained from accelerated aging tests at 350 K, closely match the R_DS_ON_ model predictions across all voltages, confirming the accuracy of (11). By comparing the experimentally measured R_DS_ON_ values with the initial R_DS_ON_ values obtained under specific conditions (350 K and V_bus_ = 80 V), a slow yet significant increasing trend for R_DS_ON_ can be observed. This variation can be attributed to the combined effects of multiple factors, including HEMT aging, V_bus_, and junction temperature, which render direct prediction of the HEMT RUL impractical. Therefore, the primary step is to precisely calibrate the HEMT R_DS_ON_ to eliminate the interference caused by junction temperature and V_bus_. Figure 10b,c show a comparison between the HEMT junction temperature changes fitted by the TSEP model using (12) and the junction temperature values measured with a high-precision platinum resistance thermometer (PRT). The margin of error is within 3%. Combining the V_bus_ values, (1) can be used to calculate the equivalent R_DS_ON_ under the conditions of a junction temperature of 350 K and V_bus_ = 80 V. During hard switching operation, hot electrons with high kinetic energies can be generated in the channel of HEMTs, particularly in low-voltage HEMTs (<200 V). These hot electrons traverse the AlGaN precursor barrier and subsequently enter the conduction band of passivation, where they become trapped in deep intermediate bandgap states within the insulator [39,40]. Once electrons infiltrate the dielectric layer, they are captured near the surface, thereby increasing the density of trapped charges at the surface. These electrons do not escape, causing a permanent increase in R_DS_ON_ due to enhanced charge trapping in the dielectric [29,41]. By gaining an understanding of the intrinsic wear-out mechanism, a physics-based lifetime model was developed based on the impact ionization mechanism [37]. This model describes how hot electrons, accelerated by high electric fields during hard-switching, are injected into the dielectric’s conduction band and trapped in deep mid-gap states, leading to a logarithmic increase in R_DS_ON_ over time. The variation in R_DS_ON_ over time is depicted in Equation (13) [37,42]:

(13) $$\frac{\Delta R_{\mathrm{DS}\_\mathrm{ON}}\left(t\right)}{R_{\mathrm{DS}\_\mathrm{ON}}\left(0\right)}=a+\mathrm{blog}(1+\exp\left(\frac{V_{\mathrm{DS}}-V_{\mathrm{FD}}}{\alpha }\right))T^{1/2}\;\exp(h\omega_{0}/\mathrm{kT})\log(t)$$

In the degradation process of GaN HEMTs, R_DS_ON_ exhibits notable regularity and trends. This makes R_DS_ON_ a valuable feature parameter for establishing a lifetime prediction model, which can greatly assist in accurately assessing the RUL of HEMTs. Equation (13), derived from the EPC reliability report [42], can be used to calculate the expected operating time of a HEMT under specific conditions. The HEMT lifetime under hard-switching conditions is defined as the time when R_DS_ON_ increases by 20% from its initial value. The HEMT RUL is then determined by adopting the following equation:

(14) $${t_{\mathrm{RUL}}=\mathrm{MTTF}}_{{(V}_{\mathrm{bus}}=80V,T=350K)}-t_{\mathrm{total}}$$

Figure 10d shows the calibrated R_DS_ON_ variation results at V_bus_ = 80 V and 350 K. To calculate the MTTF of the HEMT under specific conditions, Equation (13) can be utilized. At V_bus_ = 80 V and 350 K, the MTTF is 5 × 10^8^ min. At V_bus_ = 100 V, the MTTF is 5128 min, with an RUL of approximately 4628 min.

## 5. Conclusions

This study presents a comprehensive approach to addressing the reliability challenges of p-GaN gate GaN HEMTs, focusing on the critical issue of R_DS_ON_ increase due to electrical and thermomechanical stresses. Through the design of an optimized multiple-pulse tester board and the development of a self-calibrating TSEP model based on gate leakage current (I_G_), we achieved precise junction temperature estimation and reliable R_DS_ON_ measurement under diverse operating conditions. The proposed self-calibrating TSEP approach, utilizing I_G_ with precise calibration for aging effects, addresses a critical gap in existing research by ensuring accurate junction temperature estimation despite gate degradation, a challenge previously unaddressed in GaN HEMT reliability studies. Long-term hard-switching tests validated the model’s accuracy, with calibrated R_DS_ON_ measurements enabling robust remaining useful life (RUL) predictions, as evidenced by MTTF values ranging from 5128 min at 100 V to 5 × 10^8^ min at 80 V. These findings underscore the potential of the proposed methodology to enhance the reliability and performance evaluation of GaN HEMTs in high-power applications, such as AI power supplies and robotics, paving the way for more resilient power electronics systems.

## Figures and Tables

**Table 1:** Key components of the GaN HEMT test board.

Component	Specification	Role
Gate Driver IC	LMG1025-Q1	Reduces gate noise.
Small Signal Schottky Diode	1N6263	Monitors I_G_ via VSBD.
Current Shunt Resistor	SSDN-414-10, 0.1Ω	Measures I_DS_ON_ current.
Operational Amplifiers	LM6172IMX	High-speed test.

## Data Availability

The data that support the findings of this study are available from the corresponding author upon reasonable request.
